# Protective Effects of the Polyphenol Sesamin on Allergen-Induced T_H_2 Responses and Airway Inflammation in Mice

**DOI:** 10.1371/journal.pone.0096091

**Published:** 2014-04-22

**Authors:** Ching-Huei Lin, Mei-Lin Shen, Ning Zhou, Chen-Chen Lee, Shung-Te Kao, Dong Chuan Wu

**Affiliations:** 1 Translational Medicine Research Center, China Medical University Hospital, Taichung, Taiwan; 2 Graduate Institute of Chinese Medicine, China Medical University, Taichung, Taiwan; 3 Graduate Institute of Clinical Medical Science, China Medicine University, Taichung, Taiwan; 4 Department of Microbiology and Immunology, School of Medicine, China Medical University, Taichung, Taiwan; University of Rochester Medical Center, United States of America

## Abstract

Allergic asthma is a lifelong airway condition that affects people of all ages. In recent decades, asthma prevalence continues to increase globally, with an estimated number of 250,000 annual deaths attributed to the disease. Although inhaled corticosteroids and β-adrenergic receptor agonists are the primary therapeutic avenues that effectively reduce asthma symptoms, profound side effects may occur in patients with long-term treatments. Therefore, development of new therapeutic strategies is needed as alternative or supplement to current asthma treatments. Sesamin is a natural polyphenolic compound with strong anti-oxidative effects. Several studies have reported that sesamin is effective in preventing hypertension, thrombotic tendency, and neuroinflammation. However, it is still unknown whether sesamin can reduce asthma-induced allergic inflammation and airway hyperresponsiveness (AHR). Our study has revealed that sesamin exhibited significant anti-inflammatory effects in ovalbumin (OVA)-induced murine asthma model. We found that treatments with sesamin after OVA sensitization and challenge significantly decreased expression levels of interleukin-4 (IL-4), IL-5, IL-13, and serum IgE. The numbers of total inflammatory cells and eosinophils in BALF were also reduced in the sesamin-treated animals. Histological results demonstrated that sesamin attenuated OVA-induced eosinophil infiltration, airway goblet cell hyperplasia, mucus occlusion, and *MUC5AC* expression in the lung tissue. Mice administered with sesamin showed limited increases in AHR compared with mice receiving vehicle after OVA challenge. OVA increased phosphorylation levels of IκB-α and nuclear expression levels of NF-κB, both of which were reversed by sesamin treatments. These data indicate that sesamin is effective in treating allergic asthma responses induced by OVA in mice.

## Introduction

Allergic asthma is a chronic airway inflammatory disease that affects over 300 million people worldwide [1]. It is generally believed that asthma is developed due to allergenic stimuli that activate multiple inflammatory cells to release inflammatory mediators, causing pathophysiological changes in vascular permeability, bronchoconstriction, epithelial hypertrophy, and mucus production [2], [3]. During allergic sensitization, activation of allergen-specific T helper 2 (T_H_2) cells releases cytokines to induce B cells (via IL-4 and IL-13) and to promote eosinophil maturation (via IL-5) [4]–[6]. From B cells IgE is synthesized and secreted to recruit mast cells that release inflammatory mediators including histamine, cysteinyl leukotrienes, and cytokines [7]. In the late allergic responses, the degree of eosinophilia is highly related to the severity of asthma, suggesting that eosinophils are the principal effector cells in asthma progression [8].

At present, well-established therapies for allergic asthma mainly include inhaled corticosteroids and β-adrenergic receptor agonists. Corticosteroids take effect by suppressing the expression of inflammatory genes such as nuclear factor-κ B (NF-κB), which is activated during inflammatory responses to increase expression levels of cytokines, chemokines, and adhesion molecules [9], [10]. Inhaled β-adrenergic agonists are effective bronchodilators that mainly relax airway smooth muscle and quickly relieve asthma symptoms [11], [12]. However, there exist concerns about the long-term effects of these treatments. For instance, corticosteroids might lead to cataracts, osteoporosis in elders and stunting of growth in children [13]. Long-acting β-agonists might delay recognition of increasing inflammation [14]. Therefore, developments of new therapeutic intervention are needed as alternative or supplement to current asthma treatments.

Polyphenols are a large structural class of natural compounds ubiquitously distributed in plants sources and have long been important components of human diet. The mean daily intake of polyphenols from foods could reach a level of about 3000 mg/day [15]. Recent accumulating evidence from clinical studies and animal models has suggested that various types of polyphenols have potential protective functions against allergic diseases [16]–[19]. These polyphenols might interact with antigens to alleviate sensitization or inhibit mediator cells to reduce cytokine release [20]. The possible mechanisms of the anti-inflammatory effects of polyphenols involve the ability of metal ion complexation, macromolecule complexation, free radical scavenging, and anti-oxidation [21], [22]. In addition, one large subclass of polyphenols, termed flavonoids, are structurally similar to steroid hormones, with some members acting on certain nuclear receptors via agonist or antagonist activities [23]. Therefore, the abundant dietary sources, the relatively low toxicities, and the potential bioactivities of polyphenols may provide possible alternative options for relieving asthma-associated symptoms.

Sesamin is a flavonoid-like polyphenolic compound found in plants such as *Asarum sieboldii* [24]–[26]. It is also one of the major bioactive compounds in sesame seeds and is thought to account for the anti-oxidative properties of sesame oil [27]. It has been reported that sesamin is able to mitigate hypertension, to attenuate thrombotic tendency, and to prevent neuroinflammation and apoptotic cell death *in* *vitro* [28]–[30]. However, it remains unknown whether sesamin exhibits therapeutic effects in allergic diseases. In the present study, we aimed to investigate the influence of sesamin on allergic asthma in a murine model of OVA-induced airway inflammation and hyperreactivity. Our results revealed that sesamin decreased OVA-induced secretion of IgE and cytokines, prevented eosinophil infiltration, and attenuated mucus over-secretion, likely by a mechanism of upregulating IκB-α and inhibiting NF-κB signaling pathways.

## Materials and Methods

### Chemicals and Reagents

Sesamin (5,5′-(1S,3aR,4S,6aR)-tetrahydro-1H,3H- furo[3,4-c]furan-1,4-diylbis (1,3-benzodioxole); CAS No. 607-80-7; molecular weight 354.4; purity 98%) was purchased from Yuanye Biological Co., Ltd. (Beijing, China). Ovalbumin and Piodic acid Schiff (PAS) kits were purchased from Sigma-Aldrich (St. Louis, MO, USA). IL-4, IL-5, and IgE kits were purchased from BD Biosciences (San Diego, CA USA). IL-13 kit was purchased from R&D systems (Minneapolis, MN, USA). OVA (Sigma) for intraperitoneal (i.p.) injection was adsorbed to aluminium hydroxide adjuvant (Santa Cruz, Dallas, TX, USA) at a ratio of 50 µg to 2 mg in 200 µl PBS. OVA for intratracheal (i.t.) injection was dissolved in saline at a final concentration of 2.5 mg/ml. Sesamin was prepared in DMSO and diluted by saline plus PEG400 (final DMSO concentration <0.5%). Saline plus PEG400 (Sigma-Aldrich) with 0.5% DMSO was used as a vehicle in control groups. The final concentration of DMSO in the reaction mixture was below 0.5%.

### Animals

All mice in this study were males of BALB/c genetic background at 6–8 weeks of age obtained commercially (BioLasco co., Ltd.,Taiwan). Animals were housed under controlled laboratory conditions with a 12-h dark-light cycle. Experimental protocols of the present study were evaluated and approved by the Institutional Animal Care and Use Committee of China Medical University according to Care of the animals and surgical procedures of China Medical University Protocols.

### Asthma Model Induced by OVA Sensitization and Challenge

**Figure 1:**
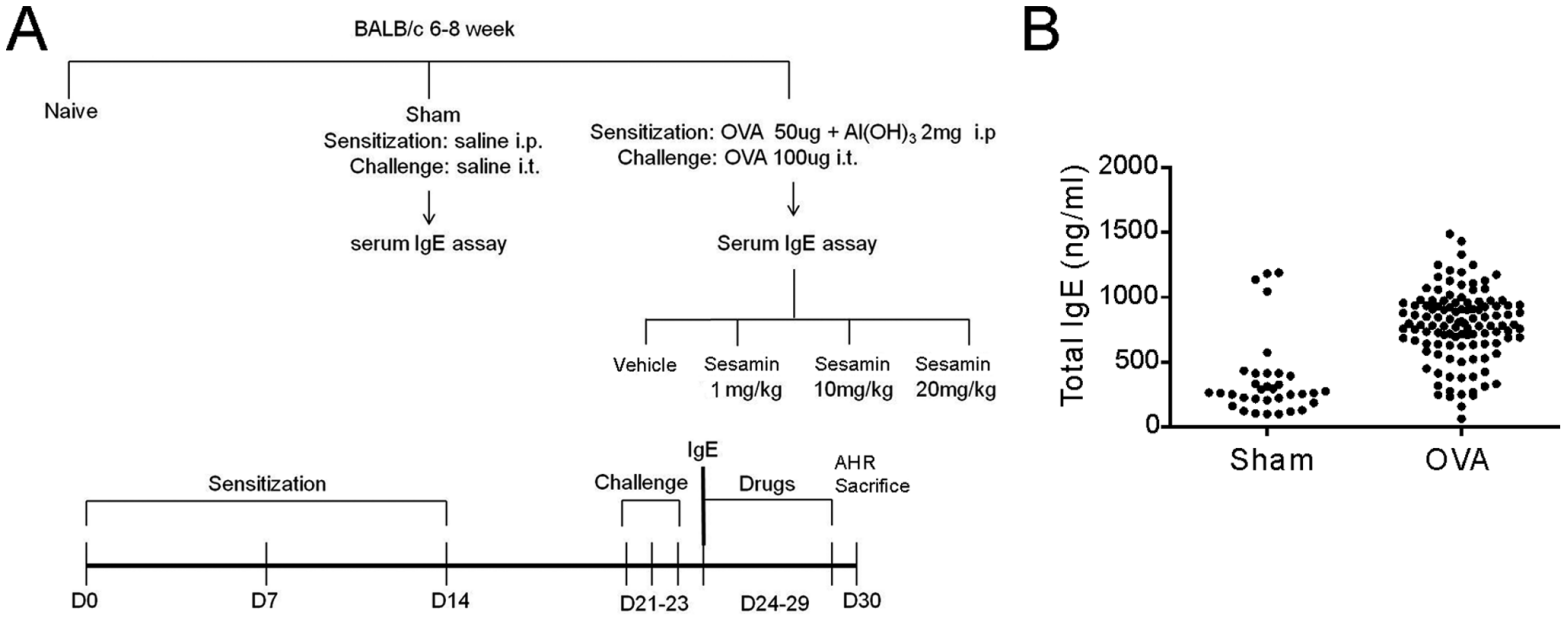
Establishment and validation of the OVA-induced asthma model. (A) Schematic flow chart of experimental design. (B) Dot plot of the serum IgE levels from blood samples collected on day 24 in the sham (n = 34) and the OVA group (n = 115). Animals from both groups with IgE level as outliers or the OVA-challenged animals with IgE level below the cutoff value (<495 ng/ml) were excluded from further experiments (see Table S1 for details). The final animal numbers used in Fig. 2 to Fig. 8 were n = 30 in the sham group and n = 80 in the OVA group. The averaged IgE levels were 264.7±21.0 and 793.1±14.6 (p<0.01) in the sham and OVA group, respectively. The final number of mice analyzed for each end point was between 5 to 11 as described in the figure legends.

The asthma model was established according to Hung *et* *al.* [31]. A schematic diagram of the treatment schedule is shown in Fig. 1A. Mice received i.p. injection of 50 µg of OVA on day 0, 7, and 14, respectively (Fig. 1A). On day 21, 22, and 23 after the first immunization of OVA, mice were challenged with i.t. instillation of OVA (100 µg) after anesthetized with isoflurane. Serum IgE levels were examined using blood samples collected from the orbital sinus plexus of surviving animals one day after the last i.t. instillation of OVA. Based on the serum IgE levels, some animals were excluded from further experiments according to the following criteria: outliers out of the outer fences, outliers out of the ELISA detection range, or the IgE values of OVA-challenged mice below the cutoff value. The upper outer fence and the cutoff value were calculated by the following equations:
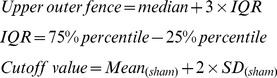



Where IQR is interquartile range and SD is standard deviation. The IgE levels of all animals were plotted in Fig. 1B (n = 34 in the sham group and n = 115 in the OVA group). In the OVA group, animals with IgE levels less than 495 ng/ml (cutoff value) were considered not to have allergic responses and were not used for further drug treatments. The final animal numbers included in experiments were n = 30 in the sham group and n = 80 in the OVA group. More detailed statistic analysis of the outliers and the cutoff value were summarized in Table S1.

The OVA-stimulated mice were subjected to subsequent i.p. injection of vehicle or of 3 different dosages of sesamin (1 mg/kg, 10 mg/kg, and 20 mg/kg body weight (b.w.) daily from the 24th to the 29th day after the first immunization of OVA. All mice were sacrificed on day 30. Bronchoalveolar lavage fluid, lung tissue, and blood samples from the venous plexus were collected.

### Bronchoalveolar Lavage Fluid (BALF) Collection and Eosinophil Count

Mice were anaesthetized and the trachea was cannulated while the thorax was gently massaged. Lungs were lavaged twice with 1 ml of saline. The recovered lavage samples were cooled on ice and were centrifuged at 1200 rpm for 10 minutes at 4°C. The supernatant was collected and stored at −80°C for ELISA assays. The pellets were obtained and resuspended in a final volume of 0.5 ml in saline, and the total inflammatory cell number was assessed by counting cells in at least five squares of a hemocytometer, after exclusion of dead cells stained by trypan blue. 0.1 ml of the resuspended BALF pellet solution was loaded onto a slide and centrifuged using a cytospin. After slides were dried, cells were fixed and stained using Liu’s stain reagents. The numbers of eosinophils in a total of 400 cells were counted in each slide based on morphology and staining characteristics [32]. All cell counts were performed by two independent blinded investigators.

### Enzyme-Linked Immunosorbent Assays (ELISAs)

IL-4, IL-5 and IL-13 contained in BALF were measured using specific mouse IL-4, IL-5 and IL-13 ELISA kits. The serum IgE level was measured using an enzyme-linked immunosorbent assay antibody. All ELISAs were performed according to the manufacturer’s directions.

### Histological Analysis

The lung tissue from upper and lower lobes was collected and immediately fixed in 4% parformaldehyde/PBS (wt/vole) at 4°C. The tissue was embedded in paraffin and cut into 2–5 µm sections, and was stained with hematoxylin and eosin. The density of total imflammatory cell in the peribronchial areas of mice from different groups was assessed as the inflammatory score by semiquantitative scales: 0 (no inflammation), none; 1, minimal (occasion ruffing with inflammatory cells); 2, mild (1–3 cells); 3, moderate (4–5 cells); 4, severe (more than 5 cells) [33]–[35]. The number of eosinophils per unit of lung tissue (2200 µm^2^) was determined by a point-counting system (Image-J; NIH Image), and interstitial and perivascular airway edema was assessed [35]. To evaluate airway goblet cells and mucus in airways, the lung sections were stain with PAS. The number of goblet cells was determined as the percentage of total airway epithelial cells in each airway examined. Mucus occlusion of the airway diameter was determined by the following scale: 0, no mucus; 1, <10% occlusion; 2, approximately 30% occlusion; 3, approximately 50% occlusion; 4, greater than approximately 80% occlusion [35].

### Total RNA Extraction

Total RNA was extracted using the TRIzol RNA Isolation system (Invitrogen Life Technologies, Carlsbad, CA, USA). 0.1 g of lung tissue was thoroughly homogenized in 1 ml TRIzol reagent with a homogenizer in tissue grinder pestle. Total RNA was isolated using a standard method according to manufacturer’s instructions. Briefly, the RNA precipitate was washed twice by gentle vortexing with 70% ethanol, collected by centrifugation at 12000 rpm, dried under vacuum for 5–10 min, dissolved in 200 ul of RNase-free water (Promega, Madison, WI, USA), and incubated for 10–15 min at 55–60°C. The RNA was quantified and checked for purity and condition by spectrophotometry at wavelength of 260 nm. The extracted integrity was assessed by 2% agarose gel electrophoresis and RNA was visualized by ethidium bromide staining.

### Reverse Transcription Polymerase Chain Reaction (RT-PCR)

The MUC5AC mRNA transcripts were measured by RT-PCR as previously described [36]. Glyceraldehyde 3-phosphate dehydrogenase (GAPDH) was chosen as the endogenous control gene. The PCR primers for mouse MUC5AC and mouse GAPDH were designed according to the published cDNA sequnces as follows: The MUC5ac were (forward) 5-AGC TAC AGT GCA ACT GGA CC-3 and (reverse) 5-GGA CAC AGA TGA TGG TGA CA-3. The GAPDH were 5-ATG GTG AAG GTC GGT GTG AAC-3 and (reverse) 5-TTA CTC CTT GGA GGC CAT GTA-3 (MDBio, Taipei, Taiwan).

### Cytosolic and Nuclear Protein Extraction from the Lung Tissue and Western Blot Analysis

Cytoplasmic and nuclear proteins from lung tissues were extracted by nuclear extraction kit (Sk-0001, Signosis, Inc., Sunnyvale, CA, USA) and experiments were performed according to the manufactures instruction. Lung tissue (20 mg) was washed with ice-cold PBS and centrifuged at 1200 rpm for 10 min at 4°C. After carefully aspirating the supernatant, tissue was homogenized with 1 ml buffer I working solution, shaken for 10 minutes, and was centrifuged at 12,000 rpm for 5 min at 4°C. The supernatant containing cytoplasmic proteins was transferred to a clean tube and stored at −70°C until used. The pellet was resuspended with Buffer II working solution (250 µl) and shaken for 2 hours at 4°C,and centrifuged at 12000 rpm for 5 minutes at 4°C. The supernatant containing nuclear extracts proteins was stored at −70°C until used.

Cytosolic or nuclear protein samples from lung tissue were suspended in sample buffers, boiled for 5 min, and separated by 10% SDS-PAGE electrophoresis. The proteins were then transferred electrophoretically to methanol-rinsed PVDF membranes (Amersham, Hybond-C Extra Supported, 0.45 Micro.) in a blotting chamber at a current of 100 V for 2 hours. The blotted membranes were blocked with 5% skim milk in TBS buffer (10 mM Tris-base, 150 mMNaCl, 0.1% Tween 20) at room temperature for 1 hour and then probed with antibodies to anti-IkB-α (Santa Cruz Biotechnology), anti-phospho-IkB-α (Santa Cruz Biotechnology), anti-lamine B1 (Santa Cruz Biotechnology), anti-β-actin (Santa Cruz Biotechnology),or anti-phospho–NF-κB (Cell Signaling Technology) at 4°C overnight. The blots were then washed 3 times with TBS buffer and incubated with appropriate horseradish peroxide-conjugated secondary antibodies at room temperature for 1 hour. The membranes were finally washed 3 times, and signals were developed using enhanced chemiluminescent (ECL) reagent (Millipore, Billerica, MA, USA) according the manufacturer’s instructions. The intensities of interested bands were quantified using ImageJ 1.45 program. The relative level of target protein is expressed as the percentage between intensity of target protein and that of marker protein from the same membrane. Samples of 3 animals were randomly selected from each group and were combined for the use in one independent experiment. The final quantification results of protein levels were analyzed from three independent experiments (total of 9 animals).

### Invasive Measurements of Airway AHR

Airway resistance was assessed as an increase in pulmonary resistance in response to increasing concentrations of aerosolized methacholine (Sigma) in anesthetized mice [37]. Briefly, mice were anesthetized with 70–90 mg/kg pentobarbital sodium (Sigma). Trachea was exposed and cannulated with an 18-gauge tracheal tube. A suture around the trachea was then tied to prevent air leak. Mice were mechanically ventilated using a computer-controlled small animal ventilator (flexiVent; Scireq, Montreal, Canada) with the following parameters: respiratory rate of 150 breaths/min, tidal volume of 10 mL/kg, inhalation:exhaustion ratio of 2∶3, and positive end-expiratory pressure of 2–3 cm H_2_O. Aerosolized PBS and increasing concentrations of methacholine (3.125, 6.25, 12.5, and 25.0 mg/ml) were delivered to the animal and readings were recorded every 4 min for 12 sec at each concentration. Pulmonary resistance was calculated using a software program (flexiVent; Scireq).

### Statistical Analysis

All results were presented as mean ± standard error of mean (S.E.M.). Statistical analysis was performed using the Prism statistical analysis program (GraphPad 6.0). Kruskal-Wallis one-way ANOVA test was used for statistical comparisons of multiple groups (> = 3). Unpaired student’s t-test or Mann-Whitney u-test were used for statistical comparisons of groups of 2 with equal or unequal variance, respectively. P<0.05 was considered significant for all test. Statistical significance was presented as * p<0.05; ** p<0.01; ***, p<0.001.

## Results

### Sesamin Inhibited OVA-induced Expression of T_H_2 Cytokines and Eosinophil Infiltration in BALF

**Figure 2:**
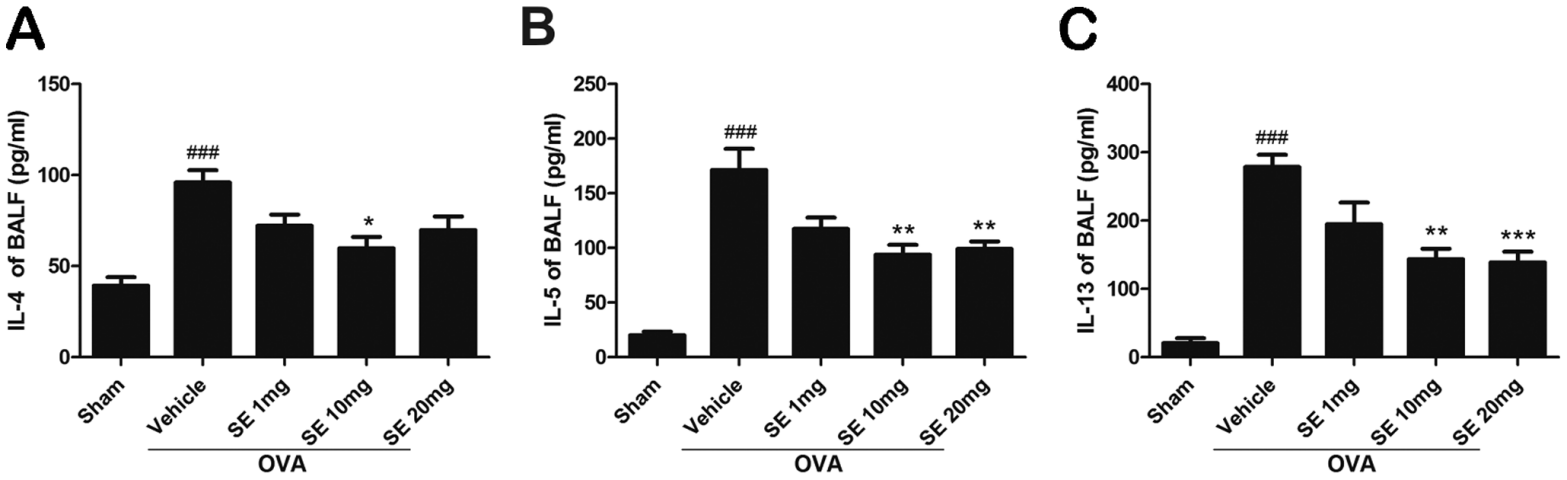
Effects of sesamin on OVA-induced expression of T_H_2 cytokines in BALF. Balb/c mice were treated with sesamin (SE, 1, 10, or 20 mg/kg b.w., i.p.) or vehicle (saline, i.p.) daily from the 24th to the 29th day after the first immunization of OVA. Expression levels of IL-4 (A), IL-5 (B), and IL-13 (C) in BALF were significantly higher in mice receiving OVA sensitization and challenge (n = 9) compared with mice receiving sham treatment (n = 5). In OVA-sensitized and challenged animals, BALF IL-4, IL-5 and IL-13 levels were inhibited in mice treated with 10 or 20 mg/kg sesamin but not with 1 mg/kg sesamin, compared with mice treated with vehicles (n = 9 for each group). Each bar represents the mean ± S.E.M. Statistical significance was defined as ^###^ p<0.01 compared with the sham group; and * p<0.05, ** p<0.01, *** p<0.001 compared with the OVA+vehicle group.

**Figure 3:**
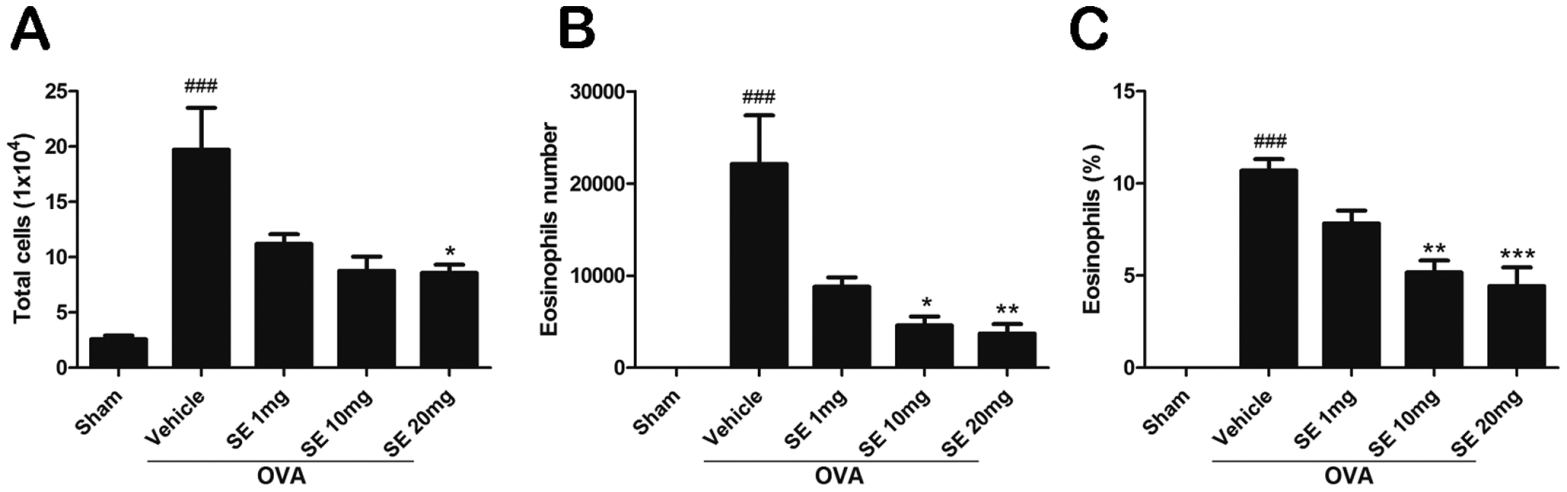
Effect of sesamin on OVA-induced recruitment of total inflammation cells and eosinophils in BALF. The number of total inflammation cells (A), number of eosinophils (B), and percentage of eosinophils in total cells in BALF were dramatically upregulated in mice receiving OVA sensitization and challenge (n = 10) compared with mice receiving sham treatment (n = 6). In OVA-sensitized and challenged animals, both total inflammation cells and eosinophils were reduced in mice treated with 10 or 20 mg/kg sesamin (n = 6 and 7, respectively) but not with 1 mg/kg sesamin (n = 6), compared with mice treated with vehicles. Each bar represents the mean ± S.E.M. Statistical significance was defined as ^###^ p<0.01 compared with the sham group; and * p<0.05, ** p<0.01, *** p<0.001 compared with the OVA+vehicle group.

The OVA sensitization and airway challenges induced remarkable increases in the levels of T_H_2 cytokines, including IL-4, IL-5, and IL-13,in BALF compared with the sham group (Fig. 2). Treatment of mice with sesamin (1, 10, and 20 mg.kg^-1^, i.p.) produced a reducing pattern of IL-4, IL-5, and IL-13 expression. Moreover, both the total cell number and the eosinophil number in BALF dramatically elevated following the OVA challenge compared with control, indicating that the OVA treatment resulted in reliable inflammatory processes in the airway (Fig. 3). The sesamin treatments resulted in a significant dose-dependent decrease in the cell count of total cells and eosinophils.

### Sesamin Reduced OVA-induce Total IgE Expression in the Serum

**Figure 4:**
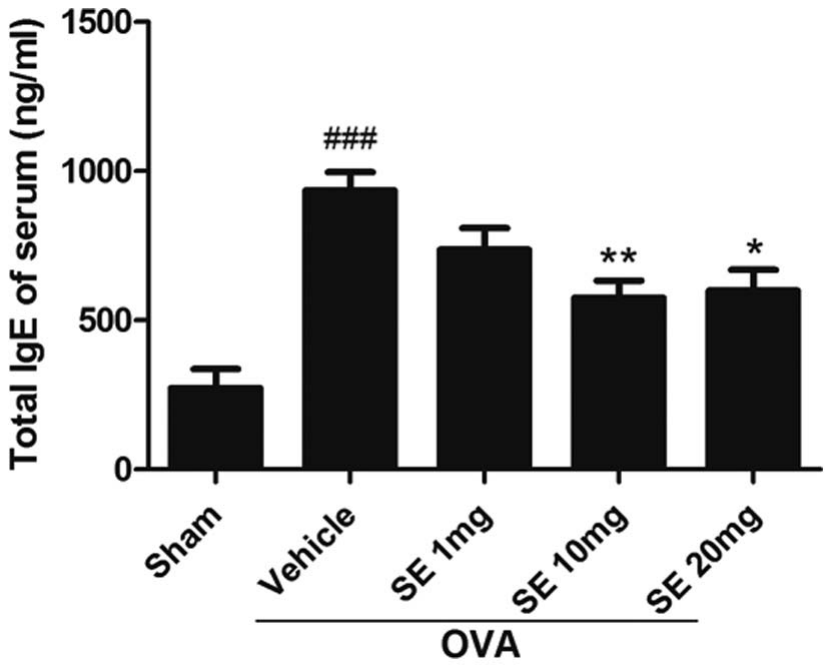
Effect of sesamin on OVA-induced upregulation of serum IgE levels. The serum level of IgE was dramatically upregulated in mice receiving OVA sensitization and challenge (n = 10) compared with mice receiving sham treatment (n = 5). In OVA-sensitized and challenged animals, serum IgE was significantly reduced in mice treated with 10 or 20 mg/kg sesamin (n = 8 and 8) but not with 1 mg/kg sesamin (n = 9), compared with mice treated with vehicles. Each bar represents the mean ± S.E.M. Statistical significance was defined as ^###^ p<0.01 compared with the sham group; and * p<0.05, ** p<0.01 compared with the OVA+vehicle group.

We examined the serum levels of total IgE, which is crucial for the development of allergic responses and is considered an important therapeutic target for allergic diseases [7]. The total IgE in the serum was significantly increased in OVA-stimulated mice compared with that in sham animals (Fig. 4). Treatments with sesamin significantly decreased total IgE levels compared with the OVA-challenged mice.

### Sesamin Attenuated OVA-induced Airway Inflammation

**Figure 5:**
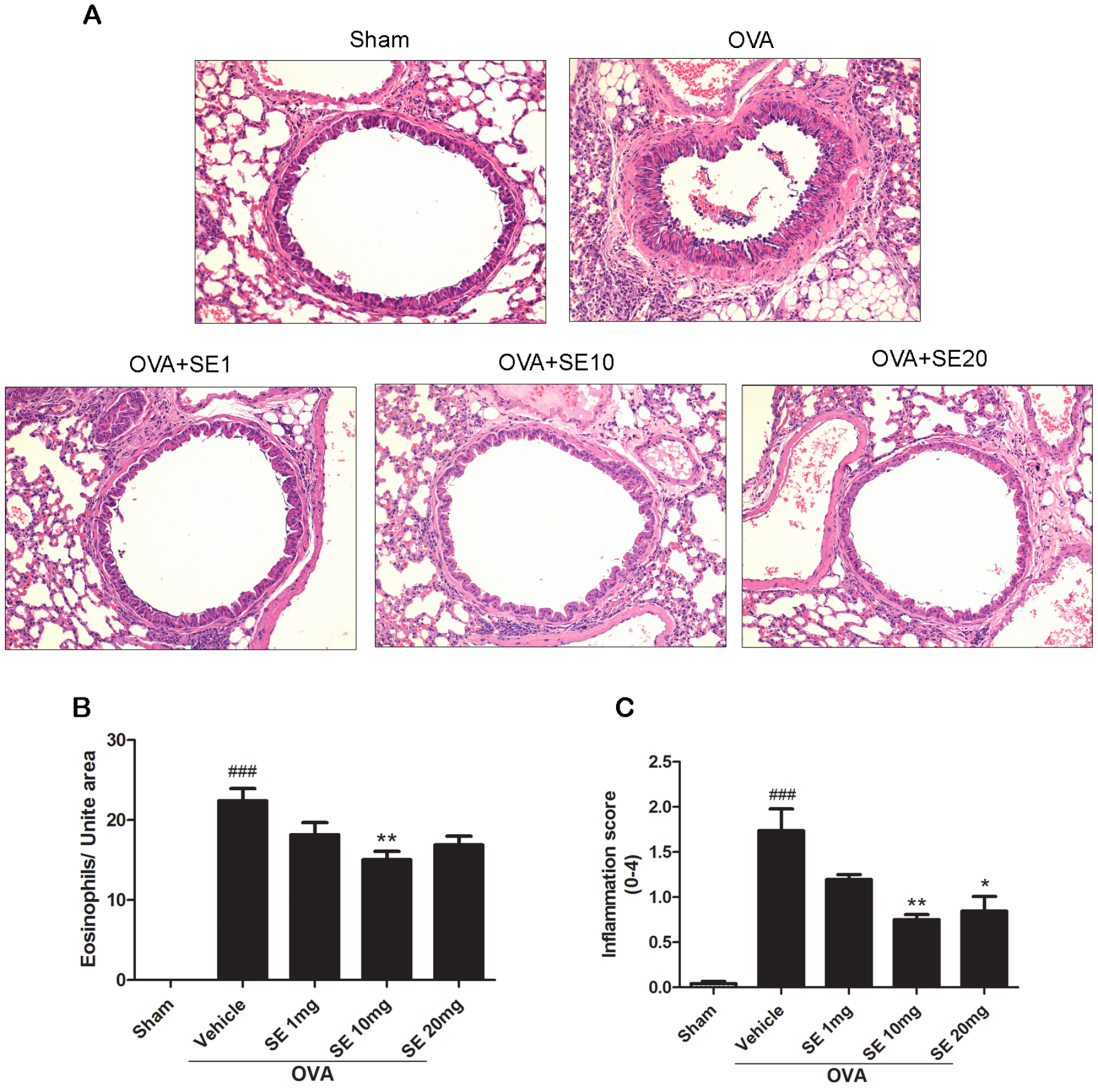
Effect of sesamin on OVA-induced lung inflammation. (A) Representative lung tissue sections from mice of sham group, OVA-sensitized and challenged mice treated with vehicle (saline, i.p.), or with sesamin (1, 10, or 20 mg/kg b.w.,i.p.). (B) The number of peribronchial eosinophils was dramatically upregulated in mice receiving OVA sensitization and challenge compared with mice receiving sham treatment (n = 9 and 9). In OVA-sensitized and challenged animals, number of eosinophils was significantly reduced in mice treated with 10 mg/kg sesamin (n = 8) but not with 1 or 20 mg/kg sesamin (n = 9 and 8, respectively), compared with mice treated with vehicles. (C) Lung inflammation was significantly attenuated in OVA-sensitized and challenged mice with 10 or 20 mg/kg sesamin (n = 8 and 8) but not with 1 mg/kg sesamin treatments (n = 9), compared with mice treated with vehicles (n = 9). Each bar represents the mean ± S.E.M. Statistical significance was defined as ^###^ p<0.01 compared with the sham group; and * p<0.05, ** p<0.01 compared with the OVA+vehicle group.

Inflammatory cells, particularly eosinophils, are dominant inflammatory cells present in the airways and greatly contribute to the pathogenesis of asthma [38], [39]. We examined lung sections from mice receiving either sham or OVA stimulation by histological assessment. Our data showed that OVA stimulation caused robust peribronchial and perivascular eosinophilia as well as interstitial and perivascular inflammatory infiltration (Fig. 5). Treatments of sesamin significantly suppressed eosinophil and inflammatory cell influx, indicating that sesamin is effective in attenuating pathological processes of asthma.

### Sesamin Inhibited Airway Goblet Cell Hyperplasia and Mucus Occlusion of the Airway

**Figure 6:**
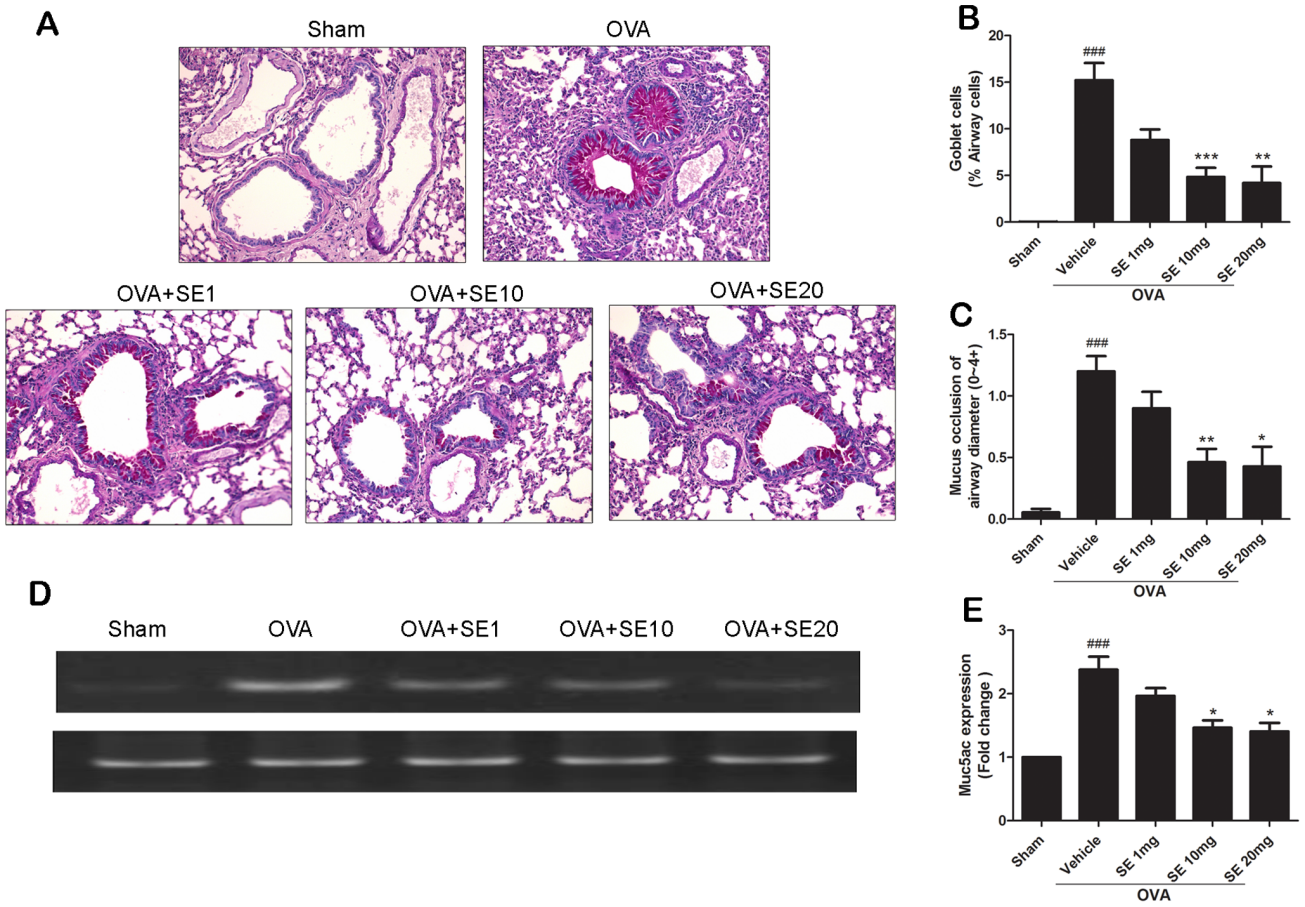
Effect of sesamin on OVA-induced airway mucus. (A) Representative lung tissue sections stained with PAS frommice of sham group, OVA-sensitized and challenged mice treated with vehicle, or sesamin (1, 10, or 20 mg/kg b.w.,i.p.). (B) The number of goblet cells was dramatically increased in mice receiving OVA-sensitization and challenge compared with mice receiving sham treatment (n = 11 and 8). In OVA-sensitized and challenged animals, number of goblet cells was significantly reduced in mice treated with 10 or 20 (n = 11 and 7, respectively) but not with 1 mg/kg sesamin (n = 8), compared with mice treated with vehicles. (C) Mucus occlusion was significantly attenuated in OVA-sensitized and challenged mice with 10 or 20 mg/kg sesamin (n = 13 and 7) but not with 1 mg/kg sesamin treatments (n = 10), compared with mice treated with vehicles (n = 9). Each bar represents the mean ± S.E.M. (D) *MUC5AC* mRNA levels were upregulated in lungs of OVA-sensitized and challenged mice and were suppressed by sesamin. (E) Relative levels of *MUC5AC* were normalized to its own GAPDH internal control and were quantified from 3 experiments. Statistical significance was defined as ^###^ p<0.01 compared with the sham group; and * p<0.05, ** p<0.01, *** p<0.001 compared with the OVA+vehicle group.

Goblet cell hyperplasia stimulates the secretion of mucus into the airway lumen and promotes airway remodeling [40]–[43]. The effect of sesamin on OVA-induce airway goblet cell hyperplasia and mucus occlusion was evaluated by staining the lung sections with PAS and assessing the percentages of stained areas. Lung sections from OVA-stimulated mice showed significant increase in mucus and goblet cells in bronchial airways compared with the sham group. In contrast, mucus and goblet cells were significantly reduced in the sesamin-treated groups (Fig. 6A–C). Moreover, the degree of mucus metaplasia was also determined by examining the mucin gene *MUC5AC* expression. The mRNA expression levels of *MUC5AC* from lung tissues of OVA-stimulated mice were significant increased compared with that of sham animals and were significantly decreased in the sesamin-treated mice (Fig. 6 D, E).

### Effects of Sesamin on Methacholine-induced Airway Hyper-Reactivity (AHR)

**Figure 7:**
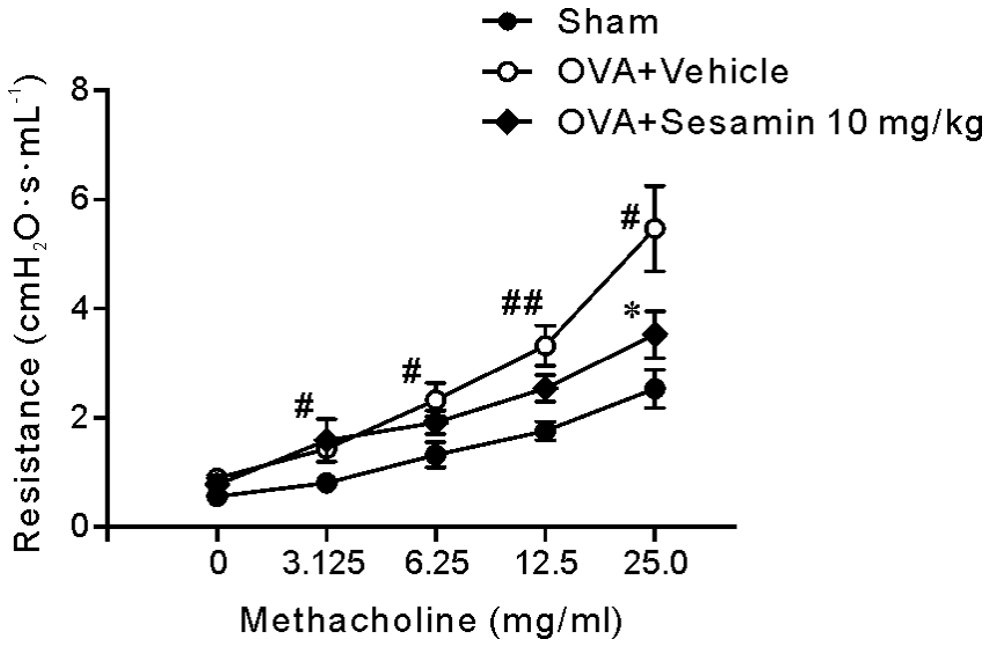
Effect of sesamin on pulmonary resistance. Pulmonary resistance in animals of the sham (n = 7), vehicle (n = 9), and 10 mg/kg sesamin groups (n = 9) showed dose-dependent AHR in response to 3.125, 6.25, 12.5, and 25.0 mg/ml methacholine. Data were presented as mean± S.E.M. Statistical significance was defined as ^#^ p<0.05 and ^##^ p<0.01 for the OVA+vehicle group compared with the sham group; * p<0.05 for the sesamin group compared with the OVA+vehicle group.

AHR was tested in sham and OVA-challenged mice treated with vehicle or 10 mg/kg sesamin by measuring airway resistance in response to methacholine on day 30 after the first immunization of OVA. Mice challenged with OVA showed significant enhanced airway resistance compared with the sham group (Fig. 7). Sesamin treatment showed significant inhibition in airway resistance compared with the control group receiving OVA challenge and vehicle treatment in response to 25.0 mg/mL methacholine, suggesting that sesamin had a protective effect against OVA-induced AHR.

### Sesamin Inhibited OVA-induced NF-κB Activation in Mice Lung Tissue

**Figure 8:**
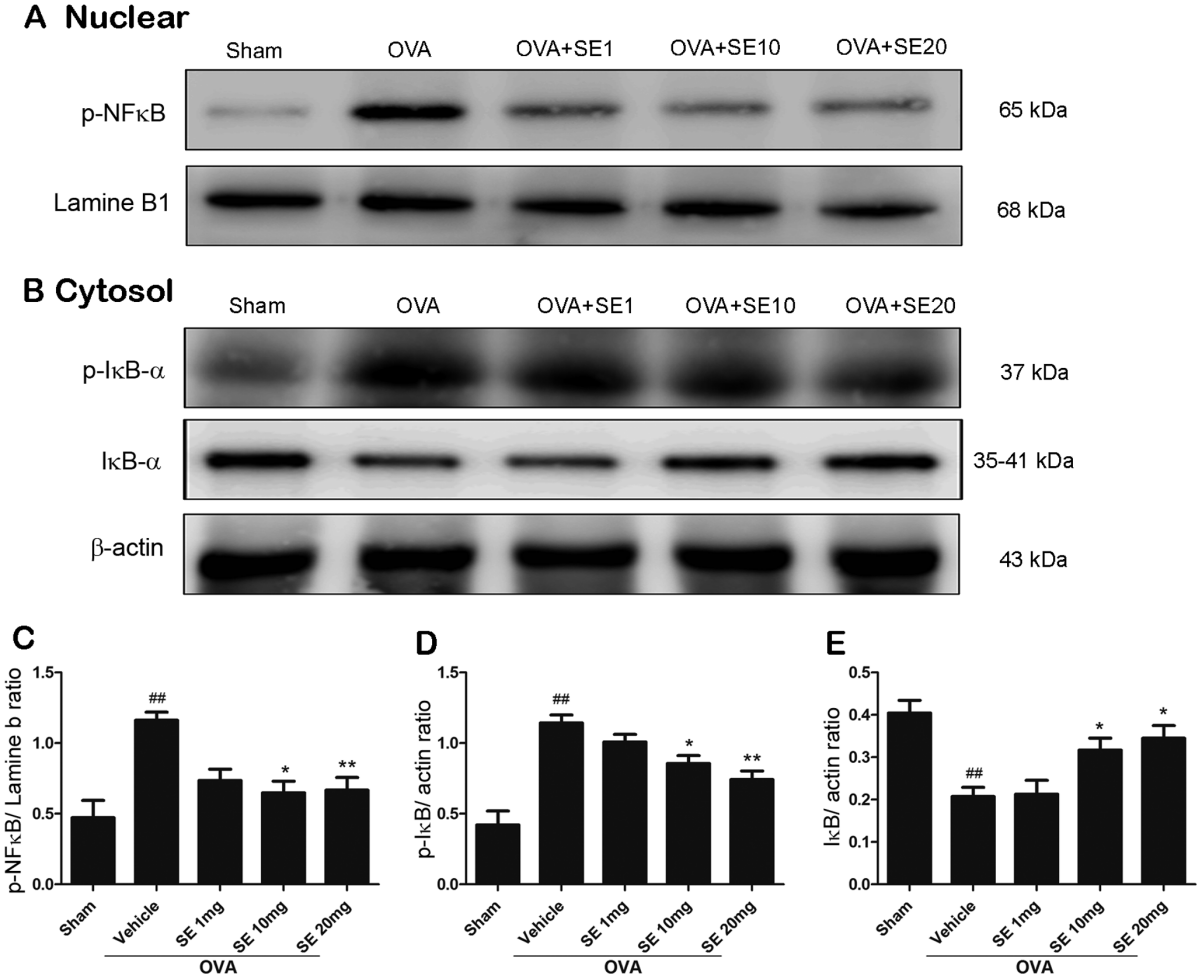
Effect of sesamin on NF-κB nuclear translocation and IκB-α phosphorylation induced by OVA. (A) and (B) Immunoblotting detection of phosphorylated NF-κB in the nucleus (A), and phosphorylated and total IκB-α (B) in the cytosol in lung tissues from mice of sham group, OVA-sensitized and challenged mice treated with vehicle, or sesamin (1, 10, or 20 mg/kg b.w., i.p.). Increased nuclear translocation of phosphorylated NF-κB and increased IκB-α phosphorylation were observed in mice receiving OVA sensitization and challenge compared with mice receiving sham treatment. Levels of total IκB-α were significantly reduced in the OVA-stimulated mice. These changes were reversed in mice treated with 10 and 20 mg/kg but not with 1 mg/kg sesamin, compared with mice treated with vehicles. The quantitative results from three independent experiments were shown in (C)-(E). Each bar represents the mean ± S.E.M. Statistical significance was defined as ^###^ p<0.01 compared with the sham group; and * p<0.05, ** p<0.01, *** p<0.001 compared with the OVA+vehicle group.

Activation of transcription factor NF-κB is required for initiating transcription of various pro-inflammatory molecules that are critical for the inflammatory responses in asthma. NF-κB is normally sequestered by the interacting protein, IκB-α, and undergoes activation and nuclear translocation with IκB-α being phosphorylated and degraded upon allergic stimulation [44]. Inhibition of NF-κB is also one of the major targets of corticosteroids, which is established to be the most effective asthma treatment currently available. Here, we tested the possibility that sesamin attenuates lung inflammation by prevention of NF-κB nuclear translocation and IκB-α phosphorylation. Our results showed that OVA-stimulation significantly increased nuclear expression levels of phosphorylated NF-κB, which were blocked by sesamin treatments (Fig. 8A, C). Remarkable increases in the phosphorylation of IκB-α were also observed in OVA-simulated animals, resulting in reduced expression of total IκB-α (Fig. 8B). The phosphorylation and degradation of IκB-α was effectively prevented in animals treated with sesamin (10 and 20 mg/kg, Fig. 8D, E).

## Discussion

### The Murine Asthma Model: Experimental Considerations

In the present study, we used the mouse model of asthma induced by OVA sensitization and challenge. OVA is one of the best-established antigen used in murine models of asthma, since it is not an environmental allergen that mice normally exposed to and it is relatively reliable to induce T_H_2 type allergic responses [45]. The initial sensitization by i.p. injection of OVA with aluminium hydroxide as an adjuvant is critical in early production of IgE in serum, which is a reliable factor in determination whether T_H_2 type allergic responses are well developed. It is noteworthy that mice exposed to OVA inhalation before i.p. sensitization resulted in suppressed serum IgE upregulation and airway hyper-responsiveness [46]. To avoid these concerns, mice used in our experiments were prevented from exposure to OVA stimuli before the i.p. sensitization procedures. Furthermore, serum IgE levels were collected from all surviving mice one day after the last OVA challenge. The animals with IgE levels below the cutoff value were considered to unsuccessfully develop T_H_2 responses and were excluded from further experiments. The insensitivity to OVA of this small percentage of mice might be due to many factors of individual differences, such as variation of endogenous corticosteroid levels that have been observed in other mice strains [47]. However, the detailed mechanism is unknown.

The allergic reactions to OVA in mouse airway typically evoke T_H_2 dominated responses with dramatically increased levels of IL-4, IL-5, and IL-13, which are accompanied by eosinophilia and IgE expression. Such a signaling cascade shares high similarities with the T_H_2 cytokine-mediated allergic responses in human asthma [7], [48], [49]. In our studies, levels of IL-4, IL-5, and IL-13 all strikingly increased in the OVA-stimulated mice, indicating that our model reliably activated the T_H_2 type of immune responses. The T_H_2 cytokine IL-5 is responsible for promoting proliferation and influx of eosinophils, whereas IL-4 and IL13 are responsible for switching B cells to IgE production [50], [51]. Our data also revealed remarkably increased eosinophilia in BALF and in lung sections as well as increased serum IgE levels. These results indicate that the murine model of asthma we studied is characteristic of T_H_2-dependent allergic airway inflammation and therefore is suitable for investigation of human asthma treatments.

### Therapeutic Effects of Sesamin on Allergic Asthma

Sesamin is one of the major bioactive components found in sesame seeds, which have long been adopted as a healthy dietary supplement in Asian foods [52]. It is well known that sesame oil has superior antioxidant properties compared with other edible oils [27]. The pharmacological effects of sesamin have been widely reported from studies of *in* *vivo* animal models, suggesting that sesamin might protect against liver damage, systemic infection, tumor induction, and hypertension [53]–[56]. Regarding its anti-inflammatory effects, the therapeutic role of sesamin in associated diseases has attracted more attention in recent years. For instance, sesamin suppressed macrophage-enhanced proangiogenesis of breast cancer cells [57]. It inhibited inflammatory responses elicited by the bacterial chemotactic peptides [58]. In an *in* *vitro* model of atherosclerosis that resembles chronic inflammation of vascular endothelial cells, sesamin reduced cytokine and adhesion molecule expression induced by low-density lipoprotein [59]. In the central nervous system, sesamin exhibited beneficial effects in kainic acid-induced epilepsy [60] and in MPP^+^-induced neuroinflammation [28].

The present study focused on the effect of sesamin on alleviating immune responses in allergic asthma. Information of allergen sensitization is normally first processed by dendritic cells, causing naïve T cells to acquire the characteristics of T_H_2 cells and releasing IL-4, IL-5, and IL-13. These cytokines are considered the central effectors of allergic inflammation and are required for induction of T_H_2-mediated airway responses [5]. In our studies, levels of IL-4, IL-5, IL-13 and IgE were all inhibited by sesamin in OVA-stimulated mice, suggesting that sesamin is effective in suppressing T_H_2 type of immune responses. IL-4 and IL-13 evoke IgE production by promoting immunoglobulin class-switch recombination in B cells. IgE diffuses into the lymphatic vessels and subsequently into the blood, binding to IgE receptors on tissue-resident mast cells [3]. Neutralization of IgE has long been considered an important target in development of asthma treatments [7]. Our data found that sesamin induced a moderate yet significant reduction in serum IgE levels, indicating an influence of sesamin on IgE-mediated reactions. Moreover, IL-5 is sufficient to induce maturation and migration of eosinophils, which are sources of multiple inflammatory proteins in epithelium damage and bronchial responsiveness, including major basic protein, eosinophil-derived neurotoxin, peroxidase, and cationic protein [61]. We showed that sesamin suppressed numbers of eosinophils in both BALF and lung tissue in OVA-stimulated mice. In addition, our histological data revealed that sesamin attenuated airway edema, hyperplasia of goblet cells, hyper-secretion of mucus, and infiltration of inflammatory cell in lung tissue, which is characteristic of pathological changes in fatal asthma in humans [61]. Taken together, our results demonstrate possible therapeutic potentials of sesamin in reducing the T_H_2 immune reaction sand the following inflammatory response mediated by IL-4, IL-5, and IL-13 in human allergic asthma.

### Possible Mechanisms of Therapeutic Effects of Sesamin

It is reported that sesamin might exert its effects by modulation of the NF-κB pathway [62], which is one of the major downstream mediator of inflammation in asthma. In quiescent cells, NF-κB is expressed in the cytosol and remains inactivated by its inhibitory binding proteins IκB-α. Following immune stimuli, the NF-κB signaling is quickly activated by various inducers, such as tumor necrosis factor alpha (TNFα), IL-1β, bacterial lipopolysaccharides (LPS), and reactive oxygen species (ROS). Binding of the inducers with pattern recognition receptors like Toll-like receptors result in phosphorylation and consequent degradation of IκB-α. Without the interaction of IκB-α, the NF-κB complex is phosphorylated and translocated in to the nucleus and initiates expression of a series of inflammation-associated gene [63]. Sesamin is reported to inhibit NF-κB activation and IκB-α degradation induced by various inducers or by upstream factors [62], [64], [65]. In line with these studies, our results demonstrated that sesamin suppressed nuclear expression of NF-κB and phosphorylation of IκB-α in lung tissues of asthma groups, indicating that sesamin prevented airway inflammation at least partially dependent upon inhibition of NF-κB pathway.

Our data showed a moderate effect of sesamin in attenuating AHR in response to methacholine administration, with significance in response to 25.0 but not the other concentrations of methacholine when compared with vehicle treatment. The relative mild effect on AHR could be explained by relatively moderate anti-inflammatory properties of sesamin. Interestingly, it remains unclear whether AHR is a causal consequence or an epiphenomenon of airway inflammation [66]. Dissociation between the two has been observed under certain pathological airway conditions [67]. In clinical studies, therapeutic treatments like long-term inhaled steroids or antibodies against interleukins may also lead to suppressed mucosal inflammation or circulating eosinophils, but did not protest against AHR and late asthmatic responses [68], [69]. Nevertheless, the moderate effects of sesamin against allergic inflammation might offer advantages when used as add-on therapy with common anti-asthma treatments.

## Conclusion

Our study has revealed that sesamin, a natural polyphenolic compound, exhibited anti-inflammatory effects to a modest but statistically significant extent in OVA-induced murine asthma model (Fig. S1). We found that treatments with sesamin significantly decreased expression levels of IL-4, IL-5, IL-13, total IgE, and numbers of eosinophils in BALF. Histological results demonstrated that sesamin attenuated OVA-induced eosinophil infiltration, airway goblet cell hyperplasia, mucus occlusion, and *MUC5AC* mRNA expression in the lung tissue. In invasive airway resistance measurements, sesamin exhibited protective effects against AHR in OVA-challenged animals. Stimulation with OVA increased phosphorylation levels of IκB-α and nuclear NF-κB, both of which were at least partially reversed by sesamin treatments. These data indicate that sesamin is effective in treating T_H_2 type allergic responses induced by OVA in the murine model of asthma.

## Supporting Information

Figure S1 **Graphic summary of anti-inflammatory actions of sesamin in the OVA-induced murine model of asthma.**
(PDF)

Table S1 **Statistics of total IgE values in animals receiving sham or OVA treatments.**
(PDF)
