# Supplementary material for: Protective Effects of the Polyphenol Sesamin on Allergen-Induced TH2 Responses and Airway Inflammation in Mice
Source: PLoS One. 2014 Apr 22;9(4):e96091. doi: 10.1371/journal.pone.0096091 (PMC3996011; doi:10.1371/journal.pone.0096091)
Supplement: Figure S1 — Graphic summary of anti-inflammatory actions of sesamin in the OVA-induced murine model of asthma. (PDF) [file pone.0096091.s001.pdf]

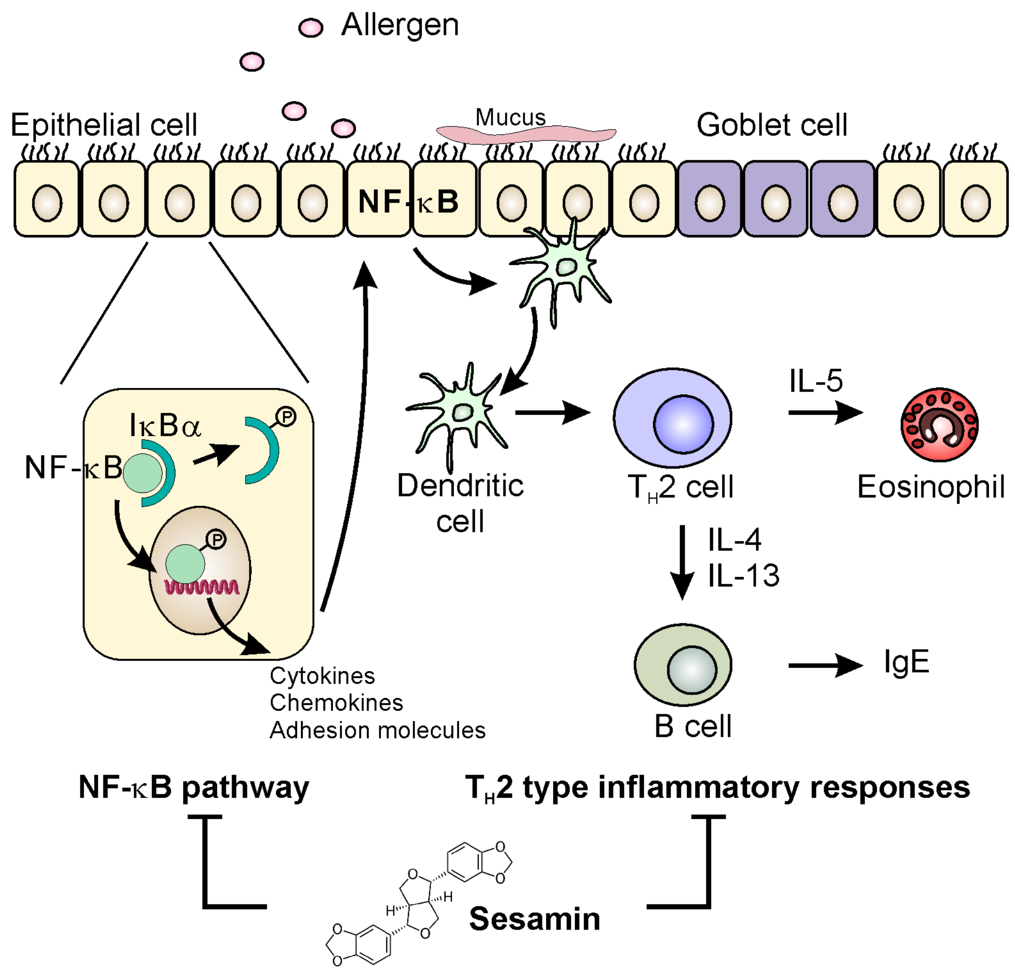

Figure S1. Graphic summary of anti-inflammatory actions of sesamin in the OVA-induced murine model of asthma. The presence of OVA allergen is sensed by dendritic cells and initiates T_H2 type inflammatory responses. T_H2 cells secrete cytokines like IL-4, IL-5, and IL-13 to induce IgE production and eosinophilic infiltration. Sesamin inhibited the upregulation of T_H2 cytokines and IgE, and suppressed accumulation of eosinophils in the airway, possibly by a mechanism of inhibition of the NF-κB signaling pathway.
