# Supplementary material for: Protective Effects of the Polyphenol Sesamin on Allergen-Induced TH2 Responses and Airway Inflammation in Mice
Source: PLoS One. 2014 Apr 22;9(4):e96091. doi: 10.1371/journal.pone.0096091 (PMC3996011; doi:10.1371/journal.pone.0096091)
Supplement: Table S1 — Statistics of total IgE values in animals receiving sham or OVA treatments. (PDF) [file pone.0096091.s002.pdf]

Table S1. Statistics of total IgE values in animals receiving sham or OVA treatments.

IgE concentrations (ng/ml) or number of animals	Groups		Notes
	Sham	OVA	
Total number of animals	34	115	Calculation of outliers [a]
Median of IgE	264.6	789.4	
Interquartile range (IQR) of IgE	211.6	353.4	
Number of outliers (> median+3×IQR)	4	0	
Number of outliers (exceeding ELISA detection range)	0	18	
Excluding the outliers			
Number of animals	30	97	Calculation of cutoff values for successful OVA challenge [b]
Passed the normality test	Yes	Yes	
Mean of IgE	264.7	707.3	
SD of IgE	115.1	225.1	
Cutoff value (the value of Mean+2SD of sham group)	-	495.0	
Number of excluded animals (IgE < cutoff value)	0	17	
Excluding the cutoff values			
Number of animals	30	80	Final data included in experiments [c]
Mean	264.7	793.1	
S.E.M	21.0	14.6	

[a] The outlier values were identified by the criteria that if the value fell out of the outer fences, where upper outer fence = median + 3 × IQR, and IQR = 75% percentile – 25% percentile.

[b] The data of both groups after excluding the outliers fit normal distribution when tested by D'Agostino and Pearson omnibus normality test. According to the normal distribution, two standard deviations (SD) from the mean account for ~95% of the data set. Therefore, we considered "$Mean_{(sham)}$+2SD" the cutoff value to determine whether an animal showed significant upregulation in total IgE levels after OVA challenge.

[c] $p<0.01$ between the sham and OVA groups after excluding the outliers and cutoff values; tested by student's t-test.
